# *Clinopodium tomentosum* (Kunth) Govaerts Leaf Extract Influences *in vitro* Cell Proliferation and Angiogenesis on Primary Cultures of Porcine Aortic Endothelial Cells

**DOI:** 10.1155/2020/2984613

**Published:** 2020-08-17

**Authors:** Irvin Tubon, Chiara Bernardini, Fabiana Antognoni, Roberto Mandrioli, Giulia Potente, Martina Bertocchi, Gabriela Vaca, Augusta Zannoni, Roberta Salaroli, Monica Forni

**Affiliations:** ^1^Grupo de investigación GITAFEC, Escuela de Bioquímica y Farmacia, Facultad de Ciencias, Escuela Superior Politécnica de Chimborazo, Riobamba. EC060155, Ecuador; ^2^Department of Veterinary Medical Science, University of Bologna, 40064 Ozzano Emilia, Italy; ^3^Department for Life Quality Studies, University of Bologna, 47921 Rimini, Italy; ^4^Grupo de Investigación Biomédica (GIB), Carrera de Odontologia, Facultad de Ciencias Medicas, Universidad Regional Autónoma de Los Andes UNIANDES, Ambato EC180150, Ecuador; ^5^Health Sciences and Technologies—Interdepartmental Center for Industrial Research (CIRI-SDV), University of Bologna, Bologna, Italy

## Abstract

*Clinopodium tomentosum* (Kunth) Govaerts is an endemic species in Ecuador, where it is used as an anti-inflammatory plant to treat respiratory and digestive affections. In this work, effects of a *Clinopodium tomentosum* ethanolic extract (CTEE), prepared from aerial parts of the plant, were investigated on vascular endothelium functions. In particularly, angiogenesis activity was evaluated, using primary cultures of porcine aortic endothelial cells (pAECs). Cells were cultured for 24 h in the presence of CTEE different concentrations (10, 25, 50, and 100 *μ*g/ml); no viability alterations were found in the 10-50 *μ*g/ml range, while a slight, but significant, proliferative effect was observed at the highest dose. In addition, treatment with CTEE was able to rescue LPS-induced injury in terms of cell viability. The CTEE ability to affect angiogenesis was evaluated by scratch test analysis and by an *in vitro* capillary-like network assay. Treatment with 25-50 *μ*g/ml of extract caused a significant increase in pAEC's migration and tube formation capabilities compared to untreated cells, as results from the increased master junctions' number. On the other hand, CTEE at 100 *μ*g/ml did not induce the same effects. Quantitative PCR data demonstrated that FLK-1 mRNA expression significantly increased at a CTEE dose of 25 *μ*g/ml. The CTEE phytochemical composition was assessed through HPLC-DAD; rosmarinic acid among phenolic acids and hesperidin among flavonoids were found as major phenolic components. Total phenolic content and total flavonoid content assays showed that flavonoids are the most abundant class of polyphenols. The CTEE antioxidant activity was also showed by means of the DPPH and ORAC assays. Results indicate that CTEE possesses an angiogenic capacity in a dose-dependent manner; this represents an initial step in elucidating the mechanism of the therapeutic use of the plant.

## 1. Introduction

Medicinal plants are presently in demand, and their acceptance is increasing progressively. According to the most recent WHO Report on Traditional and Complementary Medicine (T&CM), several countries worldwide are developing guidelines aimed at a good harmonization of T&CM therapies within their health care systems [1]. An optimal exploitation of the potential contribution of traditional medicine to the health care system was indeed identified as a key strategic objective of the “WHO Traditional Medicine Strategy: 2014-2023” [2]. In Ecuador, considered a country with a huge biodiversity, an estimated 17,000 plant species have so far been recorded, of whom more than 3,000 are medicinal plants with promising potential, widely used for healing in several cultures [3–5]. Of note, in Ecuador, current law promotes implementation of indigenous medicine in health services [1]. Despite that, most medicinal plants utilized in traditional medicine are still scientifically untested; their use is not monitored; information about effective dose, route of administration, and potential adverse effects is limited and identification of the safest, most effective and most rational practices for their use is lacking [6].

The Lamiaceae family includes thousands of aromatic plants growing in many regions of the world. Several of them have been studied for their biological and medical applications [7]; more than 50 species of this family are used to treat different affections in Ecuadorian folk medicine [8]. In particular, the *Clinopodium* genus of Lamiaceae is widely distributed in southern and southeastern Europe, Asia, and the Americas [9, 10], whereby many species of the genus are used as medicinal plants. Previous phytochemical studies on plants belonging to the *Clinopodium* genus have revealed the presence of several compounds, including flavonoids [10], phenylpropanoids [11], diterpenes, and triterpenoid saponins [12], as well as volatile and fatty oils, that exhibit different biological activities [13–15]. Seven species of *Clinopodium* are endemic in Ecuador, distributed in the central region and used, albeit not exclusively, for their medicinal properties. *Clinopodium tomentosum* (Kunth) Govaerts, commonly known as “pumin”, is a subshrub with small orange-yellow flowers that grows between 2000 and 4000 m a.s.l. In traditional medicine, local people use aerial parts of the plant to treat respiratory affections, inflammation, and gastrointestinal disorders [8]. Despite the traditional uses of the plant, at the best of our knowledge, only the phytochemical composition of the essential oil [16] and some phenolic compounds [17] have been reported. No studies regarding its biological effects *in vivo* or *in vitro* have been published.

Due to the putative anti-inflammatory effects of *C. tomentosum*, and in view of the role of angiogenesis in inflammatory response, its effects on the angiogenetic process are worth investigating. Inflammation and angiogenesis are indeed linked processes either in physiological or pathological conditions such as wound healing, tumor growth, or cardiovascular diseases [18–21]. In general, angiogenesis is tightly controlled by a balance of pro- and anti-angiogenic factors and mainly by vascular endothelial growth factor (VEGF) [22–24]. On the other hand, VEGF interacts with various cell surface receptors to mediate its cellular effects and FLK-1 (VEGFR-2) is its main signaling receptor, expressed solely by endothelial cells (ECs) [25]. Different cellular types (endothelial cells, pericytes, fibroblasts, and macrophages) and the surrounding extracellular matrix interplay a fundamental role in this complex process. In particular, ECs play a crucial role in the first step of angiogenesis mediated by tip cell migration and stalk cell proliferation [26].

It is also important to note that vascular endothelium, as a whole, is the largest organ in the body, crucial for the regulation and maintenance of the homeostasis of the whole organism [27–29]. Due to scientific interest for the multiple functions of ECs, an increasing number of publications are available on the use of their primary cultures in biological studies. Bernardini et al. [30] have isolated and characterized a primary culture of porcine aortic endothelial cells (pAECs), which have been already used as an *in vitro* model to evaluate different substances and plant extract effects on the endothelial functions [31–34].

Thus, the present research was performed with the purpose to evaluate the effects of an ethanolic extract of *C. tomentosum* (CTEE) leaves on the viability and function of vascular endothelium using pAECs. Additionally, CTEE phytochemical and antioxidant activity profiles were investigated to provide support for the possible activity mechanisms of the extract.

## 2. Materials and Methods

### 2.1. Chemicals and Reagents

Human Endothelial Serum Free Medium (hESFM), heat-inactivated fetal bovine serum (FBS), antibiotic-antimycotic (100x solution), Dulbecco's phosphate buffered saline (DPBS), and phosphate buffered saline (PBS) were purchased from Gibco Life Technologies (Carlsbad CA, USA). 3-(4,5-dimethylthiazol-2-yl)-2,5-diphenyltetrazolium bromide (MTT); Folin-Ciocalteu's phenol reagent; 1,1-diphenyl-2-picrylhydrazyl (DPPH); 6-hydroxyl-2,5,7,8-tetramethyl-chroman-2-carboxylic acid (Trolox); pure standards of phenolic acids (4-hydroxybenzoic, gallic, caffeic, chlorogenic, ferulic, *p*-coumaric, synapic, syringic, *trans*-cinnamic, and rosmarinic acids); flavonoids (quercetin, quercetin-3-*O*-glucoside, quercetin-3-*O*-rutinoside, quercetin-3-*O*-galactoside, kaempferol, kaempferol-3-*O*-rutinoside, hesperetin, and hesperidin); and HPLC-grade solvents were purchased from Sigma-Aldrich Italia (Milan, Italy). All standards (>99.5% purity in powder form) were prepared as stock solutions at 1 mg/ml in methanol and stored in the dark at -18°C for less than three months.

### 2.2. Preparation of Plant Extract

*Clinopodium tomentosum* (Kunth) Govaerts plants were collected, according to previous authorization of Ministry of the Environment (Nr. 003-IC-DPACH-MAE-2018-F), in Riobamba, Ecuador, on May 2016. Plants were identified and certified by Escuela Superior Politecnica de Chimborazo Herbarium, Riobamba, Ecuador, and a voucher specimen was deposited (N. 702). Dried leaves (300 g) were finely ground and extracted with 96% ethanol for 48 h at room temperature. After filtration, the solvent was evaporated using a rotary vacuum evaporator ( Flawil, Switzerland) and a crude extract was obtained with a yield of 5.35%. For experiments, the dry extract was dissolved in ethanol. The stock solution (20 mg/ml) was further used for HPLC analysis or diluted in culture media and membrane filtered by a 0.2 *μ*m Millipore filter (Darmstadt, Germany).

### 2.3. Cell Culture and Treatments

pAECs were isolated and maintained as previously described [30]. For this study, three biological replicates (*n* = 3), namely, cells from three different swine aortas, were employed. Briefly, cells were seeded and routinely cultured in T25 tissue culture flasks (4 × 10^5^ cells/flask) (Beckton-Dickinson, Franklin Lakes, NJ, USA). Successive experiments were conducted in 96-well plates (cell viability test), 24-well plates (wound healing migration assay), and 8-slide chambers (tube formation assay) at confluent cultures. Cells were cultured in hESFM, added with 5% FBS and 1x antibiotic-antimycotic solution in a 5% CO_2_ atmosphere at 38.5°C.

The dry extract was dissolved in ethanol (98%) to obtain a stock solution (20 mg/ml) and then in culture medium to obtain desired concentrations of CTEE and ethanol 1% for cell exposure. Ethanol (1%) was used as control vehicle.

### 2.4. Cell Viability

To determine if CTEE affects cell viability, an MTT assay was used. In particular, pAECs were seeded in 96-well culture plates at a density of 3 × 10^3^ cells/well, incubated overnight; then, media were replaced with hESFM containing 5% FBS and increasing CTEE doses (10, 25, 50, and 100 *μ*g/ml). After 24 h of incubation, MTT solution (5 mg/ml in PBS) was added to a final concentration of 0.5 mg/ml. After 2 h of additional incubation, 0.1 ml/well of MTT solubilization solution was added. Formazan Abs was determined at 570 nm, using Infinite® F50/Robotic absorbance microplate readers from TECAN Life Sciences (Männedorf, Switzerland).

The effect of CTEE on LPS-induced injury was also studied; pAECs were seeded in 96-well culture plates at a density of 12 × 10^3^ cells/well and incubated for 24 h, then exposed to different concentrations of CTEE (10, 25, 50, and 100 *μ*g/ml) in the presence of LPS (25 *μ*g/ml). After another 24 h of incubation, cell viability was evaluated by the MTT assay, as previously described.

### 2.5. Cell Cycle

When pAECs had reached 80% of confluence in a T25 flask, medium was replaced with hESFM containing 5% FBS and increasing CTEE doses (10, 25, 50, and 100 *μ*g/ml). After 24 h, pAECs were harvested, washed once in 10 ml of PBS, and 1 ml/10^6^ cells of 70% ice-cold ethanol was added drop wise with continuous vortexing. The single cell suspension was fixed at 4°C for 24 h. Then, cells were washed with 10 ml of PBS and cellular precipitate was incubated with 1 mL/10^6^ cells of staining solution containing 50 *μ*g/ml of propidium iodide (PI) and 100 *μ*g/ml of RNaseA/T1 in PBS, for 30 min in the dark at room temperature. Cell distribution in cell cycle phases was analyzed by MACSQuant® Analyzer10 and Flowlogic software (Miltenyi Biotec, Bergisch Gladbach, Germany). 2 × 10^5^ cells were examined for each sample. The Dean-Jett-Fox Univariate Model was used for this analysis [35].

### 2.6. In Vitro Wound Healing Migration Assay

To investigate CTEE's effect on pAEC migration capacity, a wound healing assay was performed as previously reported [33]. Briefly, pAECs (4 × 10^4^ cells/well) were seeded in 24-well culture plates and incubated at 38.5°C and 5% CO_2_ until confluence. Then, cells were scratched with a 200 *μ*L pipette tip along the well diameter, medium was aspirated, and the well washed twice with PBS to remove all detached cells. Cells were then incubated in hESFM with 1% FBS and increasing CTEE doses (10, 25, 50, and 100 *μ*g/ml). These culture conditions minimized pAEC proliferation. Three linear measurements of the distance between wound margins were taken for each sample immediately and 18 h after scratching. Average measurement value was an estimation of the damaged area. Images were acquired using a Nikon (Yokohama, Japan) epifluorescence phase-contrast microscope equipped with a digital camera.

### 2.7. In Vitro Capillary-Like Tube Formation Assay

The experiments were carried out using 8 -slide chambers (BD Falcon Bedford, MA, USA) coated with undiluted Geltrex™ LDEV-Free Reduced Growth Factor Basement Membrane Matrix (Thermo Fisher, Waltham, MA, USA). Extracellular matrix coating was carried out 1 h before the cell seeding in a humidified incubator, at 38.5°C, 5% CO_2_; then, pAECs (8 × 10^4^ cells/well) were seeded with increasing CTEE doses (10, 25, 50, and 100 *μ*g/ml) and incubated for 18 h. At the end of the experimental time, images were acquired using a digital camera installed on a Nikon phase contrast microscope and analyzed by Image J 64 open software (National Institutes of Health, Bethesda, USA).

### 2.8. Quantitative Real-Time PCR for VEGF and FLK-1

pAECs were seeded in a 24-well plate (4 × 10^4^ cells/well), incubated until confluence and then exposed to different CTEE concentrations (10, 25, 50, and 100 *μ*g/ml). After 24 h, cells were harvested and lysed using 1 ml Trizol reagent. A volume of 200 *μ*L of chloroform was then added to the suspension and mixed well. After incubation at room temperature (10 min), samples were centrifuged (12000 × *g* for 10 min) and the aqueous phase was recovered. An equal volume of absolute ethanol (99%) was added, and the resulting solution was applied to a NucleoSpin RNA Column. RNA was then purified according to the manufacturer's instructions. After spectrophotometric quantification, total RNA (250 ng) was reverse-transcripted to cDNA using the iScript cDNA Synthesis Kit in a final volume of 20 *μ*L.

Swine primers were designed using Beacon Designer 2.07 (Premier Biosoft International, Palo Alto, CA, USA). Primer sequences, expected PCR product lengths, and accession numbers in the NCBI database are shown in Table 1.

To evaluate gene expression profiles, quantitative real-time PCR (qPCR) was performed in CFX96 thermal cycler (Bio-Rad) using a multiplex real-time reaction (Taq-Man probes) for reference genes (GAPDH, glyceraldehyde-3-phosphate dehydrogenase; HPRT, hypoxanthine guanine phosphoribosyl transferase; and *β*-actin) and using SYBR green detection for target genes (VEGF and FLK-1, respectively). All amplification reactions were performed in 20 *μ*L and analyzed in duplicates (10 *μ*L/well). Multiplex PCR and SYBR green reactions were carried out as previously described [34]. The specificity of the amplified PCR products was confirmed by agarose gel electrophoresis and melting curve analysis. The relative expressions of the studied genes were normalized based on the geometric mean of the three reference genes. The relative mRNA expression of tested genes was evaluated as a fold of increase using the 2^-*ΔΔ*CT^ method [39] referred to pAECs cultured under standard conditions (control).

### 2.9. Total Phenol Content and Total Flavonoid Content

Total phenol content (TPC) of the extract was determined using the Folin–Ciocalteu method [40], with modifications [34]. Results, determined from regression equation of the calibration curve, were expressed as mg gallic acid equivalents (GAE)/g extract.

Total flavonoid content (TFC) was determined according to Zhishen et al. [41] with some modifications [34]. Results were expressed as mmol rutin equivalents (RE)/g extract.

### 2.10. HPLC Determination of Phenolic Acids and Flavonoids

20 *μ*L of ethanolic extract were injected into the HPLC system (Jasco, Tokyo, Japan; PU-4180 pump, MD-4015 PDA detector, AS-4050 autosampler). The stationary phase was an Agilent (Santa Clara, CA, USA) Zorbax Eclipse Plus C18 reversed-phase column (100 × 3 mm I.D., 3.5 *μ*m). The chromatographic method for the analysis of phenolic acids was adapted from Mattila and Kumpulainen [42] as detailed in Tubon et al. [34]. Gradient elution was carried out with a mixture of acidic phosphate buffer (50 mM, pH 2.5) and acetonitrile flowing at 0.7 ml/min. The signals at 254, 280, and 329 nm were used for analyte quantitation. The recovery values of phenolic acids in spiked samples ranged from 78.8 to 92.2% (RSD < 9.8%, *n* = 6). The chromatographic method for the analysis of flavonoids was adapted from Wojdyło et al. [43], as previously reported [34]. Gradient elution was carried out with a mixture of 4.5% formic acid and acetonitrile. Runs were monitored at the following wavelengths: flavan-3-ols and flavanones at 280 nm and flavonol glycosides at 360 nm. PDA spectra were measured over the wavelength range of 200−600 nm in steps of 2 nm. Retention times and spectra were compared with those of pure standards. Calibration curves were constructed for all standards at concentrations ranging from 1.0 to 100.0 *μ*g/ml (*r*^2^ ≥ 0.9998). Results are expressed as mg/g extract.

### 2.11. Antioxidant Activity Assays

Antioxidant activity (AA) of the extract was measured by the ORAC and DPPH assays. The ORAC assay was performed in an automated plate reader (Victor 3, Perkin Elmer, Shelton, CT, USA) with 96-well plates, according to Ou et al. [44] with some modifications [45]. The final ORAC values were calculated by using a regression equation between the Trolox concentration and the net area under the FL decay curve and were expressed as mmol Trolox equivalents (TE)/g extract.

The DPPH assay was done according to the method of Brand-Williams et al. [46] with some modifications. Results were determined from the regression equation of the calibration curve of Trolox in the 25-500 *μ*M range and expressed as mmol TE/g extract.

### 2.12. Statistical Analysis

The experiments were performed using three biological replicates (*n* = 3). Each treatment was replicated three or eight (viability test) times (technical replicates). Data were analysed by a one-way analysis of variance (ANOVA) followed by the *post hoc* Tukey comparison test. Differences of at least *p* < 0.05 were considered significant. Statistical analysis was carried out using GraphPad (San Diego, CA, USA) Prism 7 software.

## 3. Results and Discussion

### 3.1. Effect of CTEE on pAEC Viability, LPS-Induced Cytotoxicity, and Cell Cycle

MTT tests were carried out to evaluate if CTEE affects pAEC viability in control conditions or during an LPS-induced damage.

**Figure 1:**
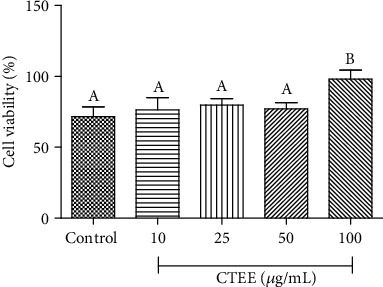
Effect of CTEE on pAEC cell viability. Cell viability was measured by the MTT assay after treatment with different concentrations of CTEE. Data represent mean ± S.D. (*n* = 3). Different letters above the bars indicate significant differences (*p* < 0.05 ANOVA post hoc Tukey's test).

After 24 h of treatment, cell viability was not affected by CTEE in the 10-50 *μ*g/ml concentration range (Figure 1), while a slight, but significant, proliferative effect was observed at the highest concentration (100 *μ*g/ml). These results are in accordance with those obtained by other authors using other species of the same genus both *in vitro* and *in vivo* [10, 47, 48].

**Figure 2:**
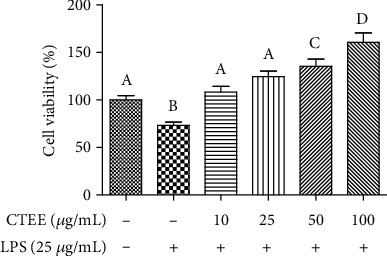
Effect of CTEE on LPS-induced pAEC damage. Cell viability was measured by the MTT assay after treatment with LPS (25 *μ*g/ml) and different concentrations of CTEE. Data represent mean ± S.D. (*n* = 3). Different letters above the bars indicate significant differences (*p* < 0.05 ANOVA post hoc Tukey's test).

Previous studies have demonstrated that primary cultures of pAECs could be used as a suitable *in vitro* model to assess not only cytotoxicity by xenobiotics, but also their effects on cell physiology, such as angiogenic and anti-inflammatory activities [31–34]. Since *Clinopodium tomentosum* is used as an anti-inflammatory agent in traditional medicine, the CTEE effect against LPS-induced injury was also investigated on pAECs by the MTT assay. LPS produced an evident cytotoxicity on pAECs, reducing their viability by about 25% (Figure 2). After 24 h of treatment with CTEE, no inflammatory damage by LPS was observed, with maximum protective effect at 100 *μ*g/ml (Figure 2).

Similar results for *Clinopodium vulgare* were reported by Burk and colleagues [9], in which the aqueous extract reduced the LPS inflammatory effect on 264.7 murine macrophages by suppressing the activation of the NF-*κ*B pathway.

**Figure 3:**
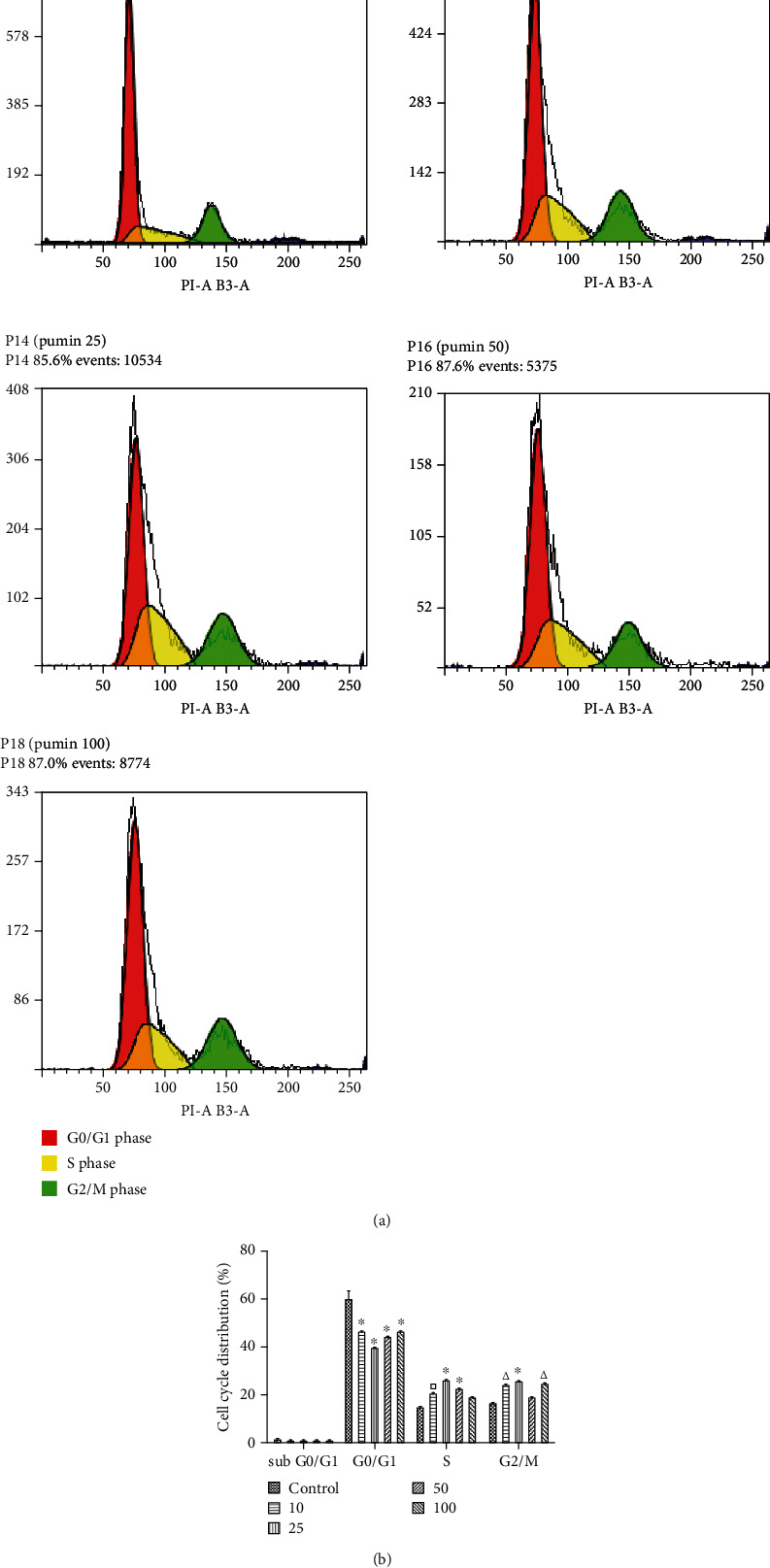
Dean-Jett-Fox Univariate cell cycle analysis by flow cytometry. Fluorescence of the PI-stained pAECs was measured using MACSQuant® Analyzer 10 and analyzed by Flowlogic software (Miltenyi Biotec, Bergisch Gladbach, Germany). 2 × 10^5^ cells were examined for each sample (*n* = 3), and the experiment was repeated twice. (a) Representative DNA content frequency histograms. (b) Cell cycle distribution for pAECs treated with various concentrations of CTEE (10, 25, 50, and 100 *μ*g/ml) for 24 h in a grouped histograms graph. (^∗^*p* < 0.0001; ^Δ^*p* < 0.001; ^□^*p* < 0.01).

Flow cytometry analysis showed that CTEE treatment affects pAEC cell cycle. As represented in Figure 3, CTEE induced a decrease in the number of cells in the G0/G1 phase and an increase in the number of cells in S and G2/M phases. Sub-G0/G1 population was essentially absent in all DNA content frequency histograms, which means that CTEE exerted a proliferative effect and did not induce either apoptosis or necrosis, at least in the tested dose range. These data are in agreement with the above described viability data.

### 3.2. Effect of CTEE on Angiogenic Activity

Considering the pivotal role of ECs in the maintenance of vascular integrity, the effect of CTEE on pAEC functional angiogenic activity was evaluated.

**Figure 4:**
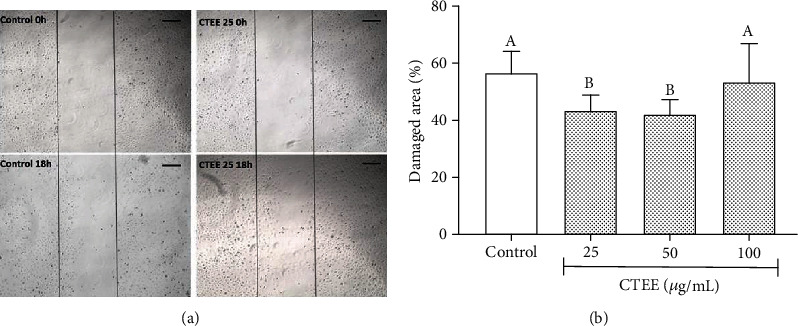
Effect of CTEE on pAEC migration capability. Cells were scratch wounded and then treated with CTEE at different concentrations (25, 50, and 100 *μ*g/ml). Photographs were recorded at 18 h after scratching. (a) Representative microscopic phase-contrast pictures showing the size of the scratch wound in the 25 *μ*g/ml CTEE-treated group compared with control. Scale bar, 100 *μ*m. (b) Damaged area (percentage of the original scratch) as a function of different CTEE concentrations. Data represent mean ± S.D. (*n* = 3). Different letters above the bars indicate significant differences (*p* < 0.05 ANOVA post hoc Tukey's test).

**Figure 5:**
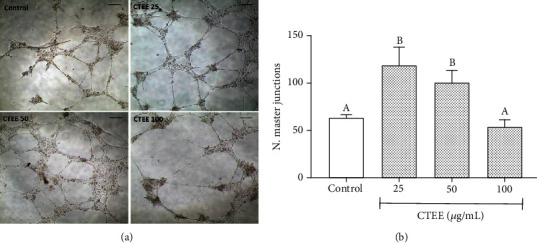
CTEE effect on pAEC tube formation capability. pAECs were cultured on an extracellular matrix for 18 h with different concentrations of CTEE (25, 50, and 100 *μ*g/ml). (a) Representative microscopic phase-contrast pictures showing capillary network in different treatment groups compared with control. Scale bar, 100 *μ*m. (b) Number of master junctions in pAEC network. Data represent mean ± S.D. (*n* = 3). Different letters above the bars indicate significant differences (*p* < 0.05 ANOVA post hoc Tukey's test).

pAEC migration capacity in a wounded edge was tested by the scratch test. Treatment with 25 and 50 *μ*g/ml CTEE significantly enhanced pAEC migration capability compared to the control; in fact, the wound area was significantly reduced. On the other hand, CTEE at 100 *μ*g/ml shared a migration capacity comparable to the control (Figure 4). Then, pAEC ability to form an organized capillary network was evaluated by *in vitro* capillary-like tube formation assay; pAECs treated with CTEE at 25 and 50 *μ*g/ml showed a significant increase in the number of master junctions compared with the control group (Figure 5). Similarly to what was seen in the migration test, pAECs treated with CTEE at 100 *μ*g/ml are not different from the control (Figure 5). These results allow to conclude that 25 and 50 *μ*g/ml CTEE treatment can improve pAEC angiogenic capability.

### 3.3. Effect of CTEE on VEGF and FLK-1 Expression

**Figure 6:**
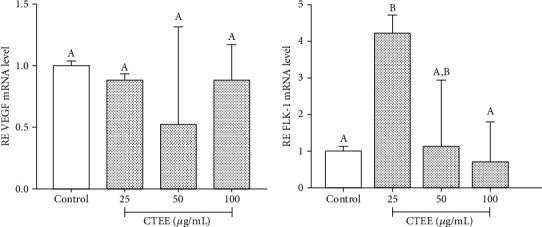
CTEE effect on VEGF and FLK-1 gene expressions. Relative expression (RE) was calculated as a fold of change with respect to the control cells; error bars represent the range of relative gene expression (*n* = 3). Different letters above the bars indicate significant differences (*p* < 0.05 ANOVA post hoc Tukey's test).

Quantitative PCR data demonstrated that CTEE significantly increases FLK-1 expression at a dose of 25 *μ*g/ml. When pAECs were treated with higher CTEE concentrations, FLK-1 expression progressively returned to basal levels. On the contrary, no significant statistical difference in VEGF mRNA expression was observed among different CTEE doses (Figure 6).

Overall, these data demonstrate that CTEE at 25 and 50 *μ*g/ml stimulated angiogenesis, but this did not occur at a higher dose (100 *μ*g/ml). In this *in vitro* model, the proangiogenic cue could be represented by a significant increase in the function of FLK-1, the main VEGF receptor.

No data regarding the angiogenic activity of *C. tomentosum* are reported in literature. However, Zeng et al. [11] showed that phenolic compounds of *Clinopodium chinensis* exerted a strong protective effect in vascular endothelial cell injury. Moreover, a protective effect against doxorubicin-induced cardiotoxicity was demonstrated both *in vitro* and *in vivo* by the flavonoid fraction of this species of *Clinopodium chinensis* [14]. Likewise, Zhang et al. [49], following the previously cited studies, demonstrated that pretreatment with a flavonoid-enriched fraction from *Clinopodium chinensis* protected against ischemic myocardial injury both *in vitro* and *in vivo*.

### 3.4. Phytochemical Analysis and In Vitro Antioxidant Capacity of CTEE

Determination of TPC and TFC revealed that CTEE contained high amounts of polyphenols (140.15 ± 0.12 mg GAE/g extract), and a relevant percentage of polyphenolic structures was represented by flavonoids (Table 2). According to HPLC-DAD analyses, rosmarinic acid was the main phenolic acid detected in the extracts, followed by chlorogenic acid and cinnamic acid, respectively. Within the flavonoid class, the flavanone glycoside hesperidin turned the most abundant compound, followed by hesperetin, kaempferol, and rutin, respectively. Moreover, CTEE showed a good antioxidant activity, as a results from both DPPH (3.72 mmol TE/g) and ORAC (4.14 mmol TE/g) assays, as shown in Table 2.

TPC in CTEE resulted very similar to that reported for a similar ethanolic extract of *Salvia sagittata*, a species belonging to the same family [34], and was of the same order of magnitude as that reported in a *Thymus vulgaris* methanolic extract [50].

Based on the phytochemical composition and the major compounds found in CTEE, it is not possible to attribute to a single molecule the effects it exerts on pAECs. Different studies confirm the protective and anti-inflammatory effect of rosmarinic acid [51–53], as well as the protective role of hesperidin in cardiovascular diseases and its angiogenic effects in diabetic foot ulcer [54–56]. Even though the role of phenolic acids on cell migration and angiogenesis has not been completely clarified and appears somehow controversial [57], an angiogenic effect has been reported for some members of the polyphenol family [58]. An increase in endothelial cell migration, accompanied by cytoskeletal reformation, was observed in an *in vitro* wound healing assay by a phenolic acid extract enriched in chlorogenic acid [59]. Thus, it is possible to hypothesize that some of the main components of CTEE, belonging to the polyphenol family, could be, at least in part, responsible for the observed angiogenic effect, even though an overall action of the whole phytocomplex cannot be excluded, given the multicomponent nature of the tested extract.

To summarize, the present study demonstrates the angiogenic effect of CTEE on pAECs but only at the intermediate doses utilized, probably mediated by the VEGF-FLK-1 pathway. However, the highest CTEE dose (100 *μ*g/ml) increased EC viability (and metabolic activity), not only in basal conditions but also in the presence of a proinflammatory stimulus. We can speculate that 100 *μ*g/ml CTEE induced stalk cell proliferation but not tip cell migration as confirmed by the absence of capillary formation capacity.

This is the first study providing a scientific rationale for the use of *C. tomentosum* in Ecuadorian traditional medicine. Nevertheless, further investigations should be performed to elucidate the pathways by which the plant extract exerts its effects on endothelial cells.

## Figures and Tables

**Table 1:** Primers used for quantitative real-time polymerase chain reaction analysis.

Gene	Sequence (5′-3′)	PCR product (bp)	Gene bank accession number	Reference
VEGF	For: CGCTCCCGAATGAACAC Rev: GCTCCTGCACCTCCTC	101	AF318502	[36]
FLK-1	For: AACGAGTGGAGGTGACAGATTG Rev: CGGGTAGAAGCACTTGTAGGC	104	AJ245446	[37]
GAPDH	For: ACATGGCCTCCAAGGAGTAAGA Rev: GATCGAGTTGGGGCTGTGACT Probe: HEX-CCACCAACCCCAGCAAGAGCACGC-BHQ1	106	NM_001206359	[38]
HPRT	For: ATCATTATGCCGAGGATTTGGAAA Rev: TGGCCTCCCATCTCTTTCATC Probe: Tx-red-CGAGCAAGCCGTTCAGTCCTGTCC-BQ2	102	NM_001032376	[34]
*β*-ACT	For: CTCGATCATGAAGTGCGACGT Rev: GTGATCTCCTTCTGCATCCTGTC Probe: FAM-ATCAGGAAGGACCTCTACGCCAACACGG-BHQ1	114	KU672525.1	[38]

**Table 2:** AA, TPC, TFC, phenolic acids, and flavonoid content in CTEE. Data are the mean ± S.E. of three technical determinations.

Assays or compounds^∗^		Concentration^∗∗^ referred to	
		Ethanolic extract	Plant dry weight
AA	ORAC	4.14 ± 0.24	0.22 ± 0.04
	DPPH	3.72 ± 0.11	0.20 ± 0.01
TPC		140.15 ± 0.12	7.50 ± 0.4
TFC		97.38 ± 0.62	5.21 ± 0.2
Phenolic acids	RA	37.03 ± 0.58	1.98 ± 0.1
	CHA	1.66 ± 0.35	0.08 ± 0.01
	CA	0.65 ± 0.03	0.04 ± 0.01
Flavonoids	HESD	16.76 ± 0.89	0.90 ± 0.03
	HEST	4.14 ± 0.14	0.22 ± 0.01
	KMP	3.97 ± 0.25	0.21 ± 0.02
	RUT	1.95 ± 0.06	0.10 ± 0.01

^∗^RA = rosmarinic acid; CHA = chlorogenic acid; CA = cinnamic acid; HESD = hesperidin; HEST = hesperetin, KMP = kaempferol; RUT = rutin. ^∗∗^units: ORAC and DPPH, mmol TE/g; TPC, mg GAE/g; TFC, mmol RE/g. phenolic acids and flavonoids, mg/g.

## Data Availability

The original data used to support the findings of this study are available from the corresponding author upon request.
